# Insights Into Stria Vascular Function, Stria Immunity, and Age-Related Hearing Loss

**DOI:** 10.1007/s10162-025-01010-2

**Published:** 2025-09-27

**Authors:** Xiaorui Shi

**Affiliations:** Department of Otolaryngology/Head & Neck Surgery, Oregon Hearing Research Center, Oregon Health & Science University, Portland, OR 97239 USA

**Keywords:** Stria vascularis, Blood-labrinth barrier, Pericyte, Perivascular macrophage, Aging-related hearing loss

## Abstract

The microvasculature plays a crucial role in maintaining auditory health by delivering essential nutrients such as glucose, ions, growth factors, and hormones, while also facilitating the elimination of metabolic waste. Simultaneously, the innate immune system acts as a protective barrier against cochlear damage caused by infections, toxic substances, and foreign agents. The normal functioning of these two systems creates an appropriate microenvironment that supports the health of sensory hair cells and spiral ganglion neurons. Disruptions in blood flow or inappropriate activation of the immune response can result in cochlear hypoxia and inflammation, both of which are linked to various auditory disorders. Understanding the characteristics and functions of these two systems could offer valuable insights into their distinct roles, potentially leading to the development of new treatments for hearing disorders associated with their dysfunction. This review covers the cellular characteristics and functions of both the vascular network and the innate immune cells within the stria vascularis, with a particular focus on how changes in both systems contribute to age-related hearing loss (ARHL), a common sensory deficit affecting the elderly population.

## Introduction

The vascular and innate immune systems within the stria vascularis (SV) are essential for maintaining cochlear homeostasis, which is crucial for optimal auditory function. The cochlea is supplied by a rich network of blood vessels located in the cochlear lateral wall, which is monitored by the innate immune system [1]. These two systems are intricately connected and functionally linked; disruptions in either can lead to various hearing disorders.

The lateral wall is primarily composed of two structures: the SV and the spiral ligament. The SV, often considered as the “powerhouse” of the cochlea, plays a vital role in generating the endocochlear potential (EP) and maintaining the proper ionic environment necessary for hearing [2, 3]. Adjacent to the SV, the spiral ligament is a fibrous structure that provides mechanical support to the SV and facilitates the transport of potassium ions (K +) through gap junctions formed by connexins Cx26 and Cx30, which are present in various types of fibrocytes [4].

**Fig. 1:**
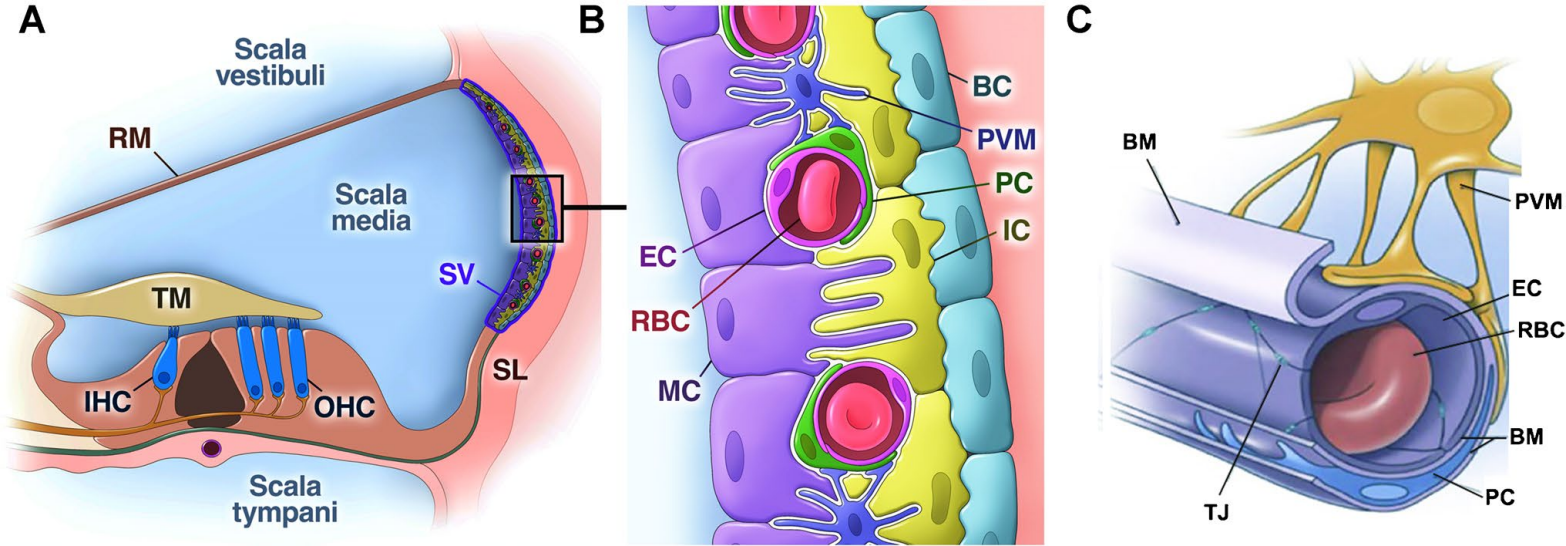
**A** Illustration of a cross-section of the cochlea, highlighting the locations of the stria vascularis and the organ of Corti. **B** The intra-strial fluid-blood barrier (ISFBB) is “sealed” or sandwiched between a layer of marginal cells (MC) and a layer of basal cells (BC). **C** This barrier consists of endothelial cells (ECs) that are interconnected by tight junctions (TJs) and is further supported by a secondary layer of pericytes (PCs) and perivascular macrophages (PVMs). *RM*, Reissner’s membrane; *TM*, tectorial membrane; *SV*, stria vascularis; *SL*, spiral ligament; *IC*, intermediate cell; *MC*, marginal cell; *BC*, basal cell; *BM*, basement membrane

The SV consists of three layers of cells organized from medial to lateral: the marginal cell layer, the intermediate cell layer, and the basal cell layer (Fig. 1A). A highly specialized capillary network is situated between the marginal and basal cell layers (Fig. 1B). Over the years, studies have demonstrated that the capillary network is populated by rich pericytes (PCs) and surrounded by a significant population of tissue-resident macrophages, known as perivascular resident macrophages (PVMs, Fig. 1C) [1]. These immune cells patrol the capillary network and protect against infections, toxic substance damage, and injuries in the cochlear SV. The interactions between the vascular and immune systems are essential for ensuring energy is sufficiently delivered to the cochlea and for the removal of harmful metabolites and toxic substances. While the cellular communication and molecular signaling between these two systems have not been extensively studied, their unique structural interactions highlight their potential role in maintaining a healthy microenvironment for sensory hair cells and supporting normal hearing. In fact, dysfunction in vascular function and abnormal immune cell activity has often been linked to various forms of hearing loss, including age-related hearing loss, noise-induced hearing loss, ototoxicity, and certain genetic disorders [5]. This review provides the current progress on the pathophysiology of the two systems, including their cellular characteristics and functions which particularly address how the systems dysfunctions associated with age-related hearing loss (ARHL), the most common hearing disorder among the aging population.

## Characteristics of Vascular Cells and Tissue Resident Macrophages in the SV

### Endothelial Cells Form a Special Intrastrial Fluid-Blood Barrier (ISFBB) in the SV

**Fig. 2:**
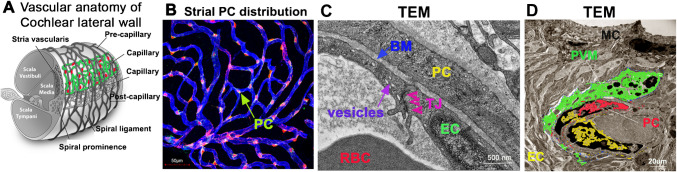
**A** An illustration of the microvascular system in the cochlear lateral wall, highlighting the microvascular networks that contain PCs in the stria vascularis. **B** A confocal projection image shows PCs labeled with PDGFRβ/tdTomato (red), distributed along strial vasculature (stained with lectin, blue). **C** A transmission electron microscopy (TEM) image shows a PC located adjacent to EC, which is rich in vesicles and connected to another EC by tight junctions (TJs), surrounded by the basement membrane (BM). **D** A TEM image illustrates the anatomical interactions among the barrier component cells: ECs, PCs, and PVM

Endothelial cells (ECs) display remarkable heterogeneity in structure and function, with site-specific characteristics and organ-specific functions [6]. The ECs in the SV possess unique characteristics that differentiate them from those in other cochlear regions, as highlighted in green in Fig. 2A. They line strial blood vessels, lack fenestrations, and exhibit high levels of tight junction (TJ) proteins such as occludin, claudins, and vascular endothelial cadherin (VE-cadherin) [7]. Mass spectrometry analysis previously by Yang et al. [8] found that these ECs contain about 40% transport proteins. These features establish the strial endothelium as a highly selective barrier, known as the interstitial fluid-blood barrier (ISFBB) [9, 10], a component of blood-labyrinth barrier (BLB) between blood and inner ear fluid (perilymph and endolymph) [11]. This barrier is supported by PCs (Fig. 2B) and a collagen IV-rich basement membrane (Fig. 2C), with PVMs present for immune surveillance (Fig. 2D) [1]. The ISFBB regulate the two-way exchange of material between the blood and cochlear lateral wall with permeability controlled by two major pathways including para-cellular and transcellular pathways [10]. Earlier studies have been shown that tightness of ISFBB barrier is linked to the expression of tight junction proteins [7] and various transporters that facilitate specific molecule movement across the barrier[8].

**Fig. 3:**
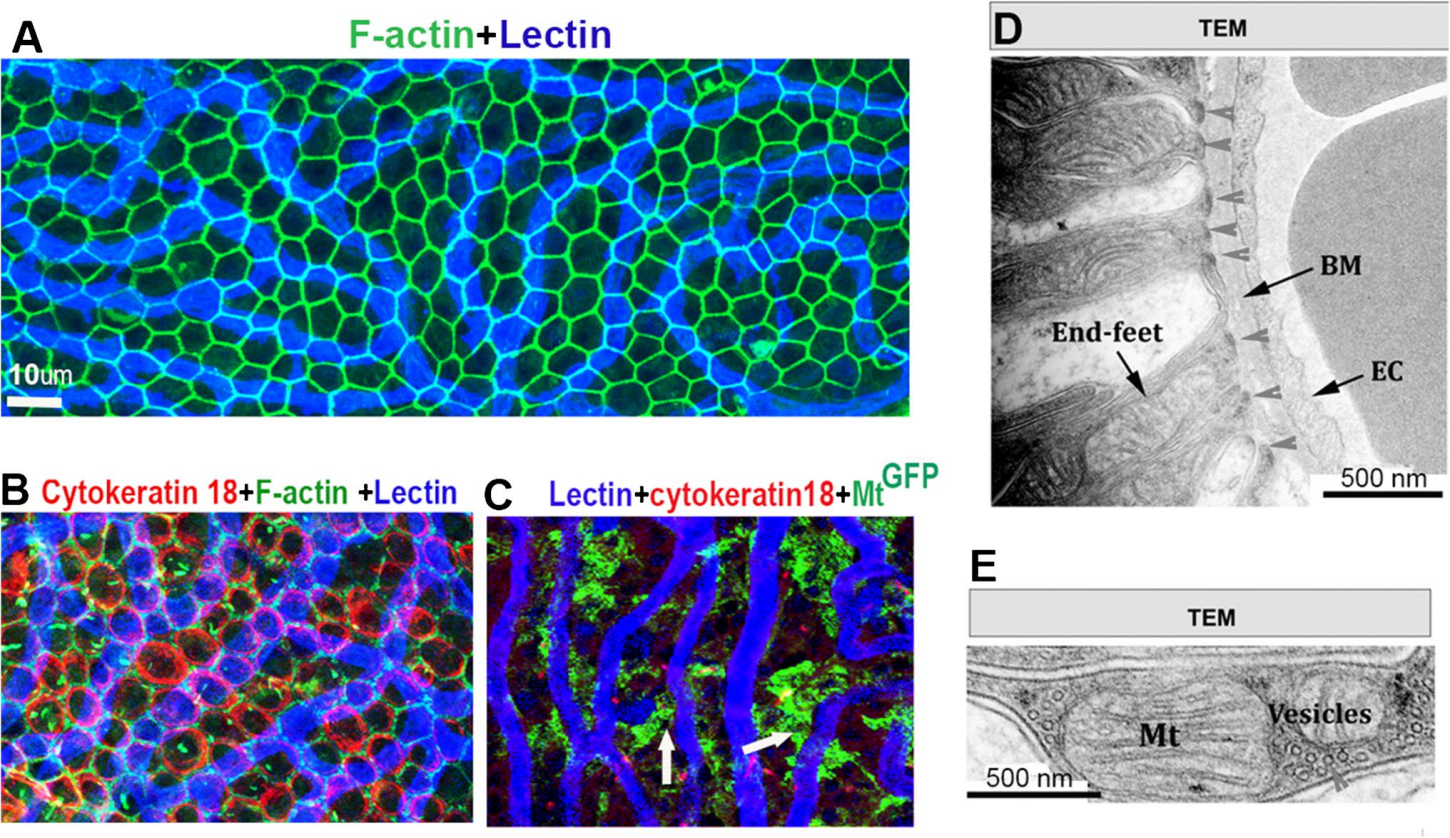
**A** Strial blood vessels, labeled with lectin (blue), located beneath the marginal cells, marked by phalloidin (green). **B** The marginal cell layer, which is labeled with both phalloidin (green) and cytokeratin 18 (red), covering a capillary network also labeled with lectin (blue). **C** GFP-tagged mitochondria within the marginal cells, indicated by cytokeratin 18 (red), which are in contact with the vessel walls in a z-section. **D** A transmission electron microscopy (TEM) image reveals the ultrastructure of the marginal cell processes (or end-feet), which contain mitochondria and vesicles in contact with endothelial cells (ECs). **E** At higher magnification, shows unidentified vesicles located on the marginal cell processes

In addition to their roles in blood supply and forming barrier structures, early and recent studies have pointed out that ECs can function as an “endocrine” gland. They actively communicate with neighboring cells to regulate organ-specific functions [12]. A recent review particularly highlights the role of the endothelium in regulating the activity of adjacent cells and managing tissue metabolic processes through the various signaling molecules they secrete in non-auditory organs [13]. However, there is limited information on how strial ECs regulate strial function by communicating with neighboring cells. An early study intriguingly showed the structural relationships between intermediate cells and capillaries in the SV of gerbils [14]. Specifically, using transmission electron microscopy, they found that certain regions between intermediate cells and EC lacked a basal lamina and showed gap junction-like membrane associations between the two cell types [14]. Additionally, they demonstrated that intermediate cells are dye-coupled with both ECs and PCs [15]. This presence of gap junctions suggests that ECs may have functions beyond simply providing a structural pathway for blood circulation. Our early ultrastructural studies intriguingly show that the processes (end-feet) of marginal cells make contact with the endothelium (Figs. 3C and D), although the nature of these contacts remains unclear. Notably, the contact areas contain a significant number of unidentified vesicles (Fig. 3E), indicating potential marginal cell communicated with ECs through those unknown vesicles.

Latest single-cell RNA sequencing (sc-RNA-seq) of the endothelial translatome in the brain, lungs, and heart indicates that ECs express signaling molecules and specific transporters that may facilitate communication with surrounding cells [16]. However, the transcriptional profile of strial ECs in the cochlea has not yet been fully characterized. Currently, most sc-RNA-seq studies of the cochlear lateral region mainly focus on strial cell types, such as marginal cells, intermediate cells, and basal cells, and there has been limited profiling of ECs due to the insufficient number of available cells. Future studies examining the gene profiles of strial ECs will be crucial for understanding their overall function within the SV, extending beyond their structural roles in blood vessels.

### PCs and Potential PC Progenitors Present in the SV Are Essential for Maintaining Vascular Stability, Permeability, and Regeneration

**Fig. 4:**
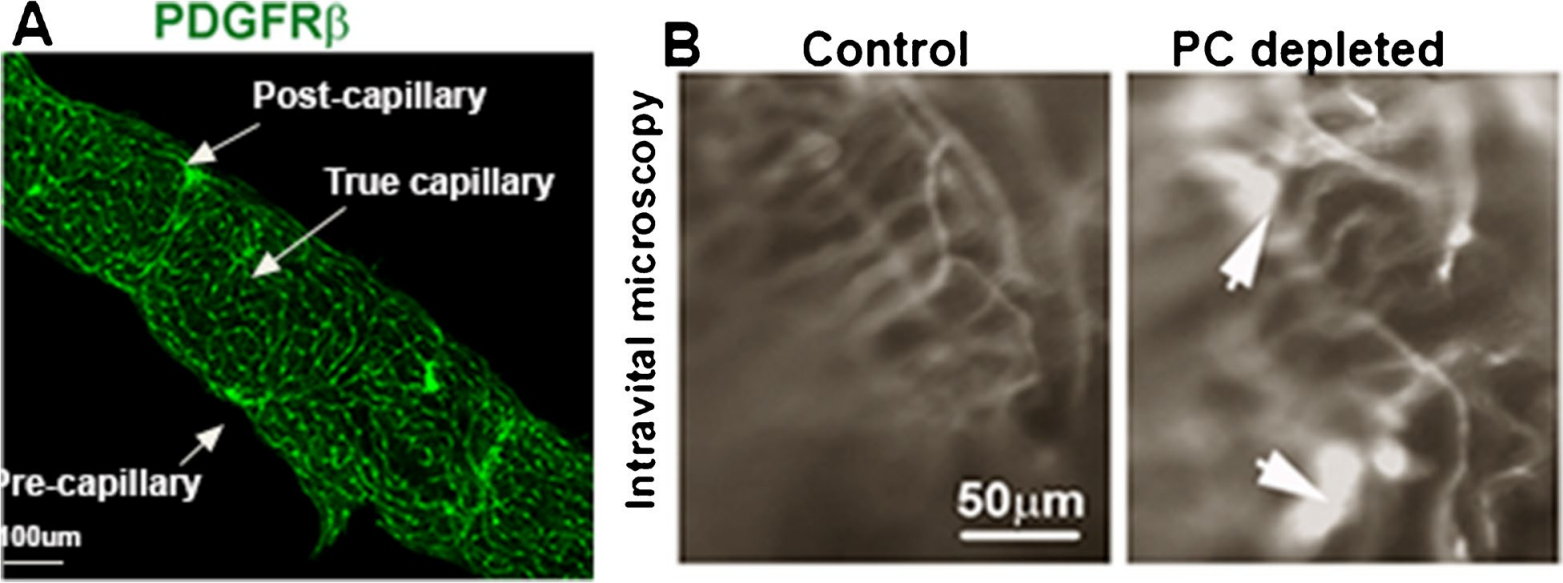
**A** A confocal projection image shows the distribution of pericytes (PCs) labeled with PDGFRβ, present in pre-capillaries, post-capillaries, and true capillaries within the stria vascularis. **B** (right) Blood flow recordings from an intravital fluorescence microscope show that PC depletion leads to vascular leakage compared to the control group with intact PCs (**B**, left)

PCs are mural cells embedded within the vascular system, primarily surrounding capillaries and microvessels where they closely interact with endothelial cells [17]. Cochlear PCs are located in the pre-capillary arterioles, capillaries, and post-capillary venules of the SV (as shown in Fig. 4A) [18]). Over the decades, research has shown that PCs in different organs are crucial for vascular development, regulation of blood flow, maintenance of vascular integrity, angiogenesis, and tissue fibrogenesis in various organs [5]. Recent research indicates that strial PCs control vascular integrity, new vessel growth, and the repair of damaged blood vessels [5]. For instance, studies using in vitro 2D co-culture methods of E Cs and PCs have demonstrated that PCs are essential for maintaining the integrity of the ISBFF by regulating TJ protein expression [7]. On the other hand, an in vivo study using a conditional PC-deficient animal model showed that the depletion of cochlear PCs leads to significant leakage in the strial ISFBB and vascular degeneration as shown in Fig. 4B, left [19].

**Fig. 5:**
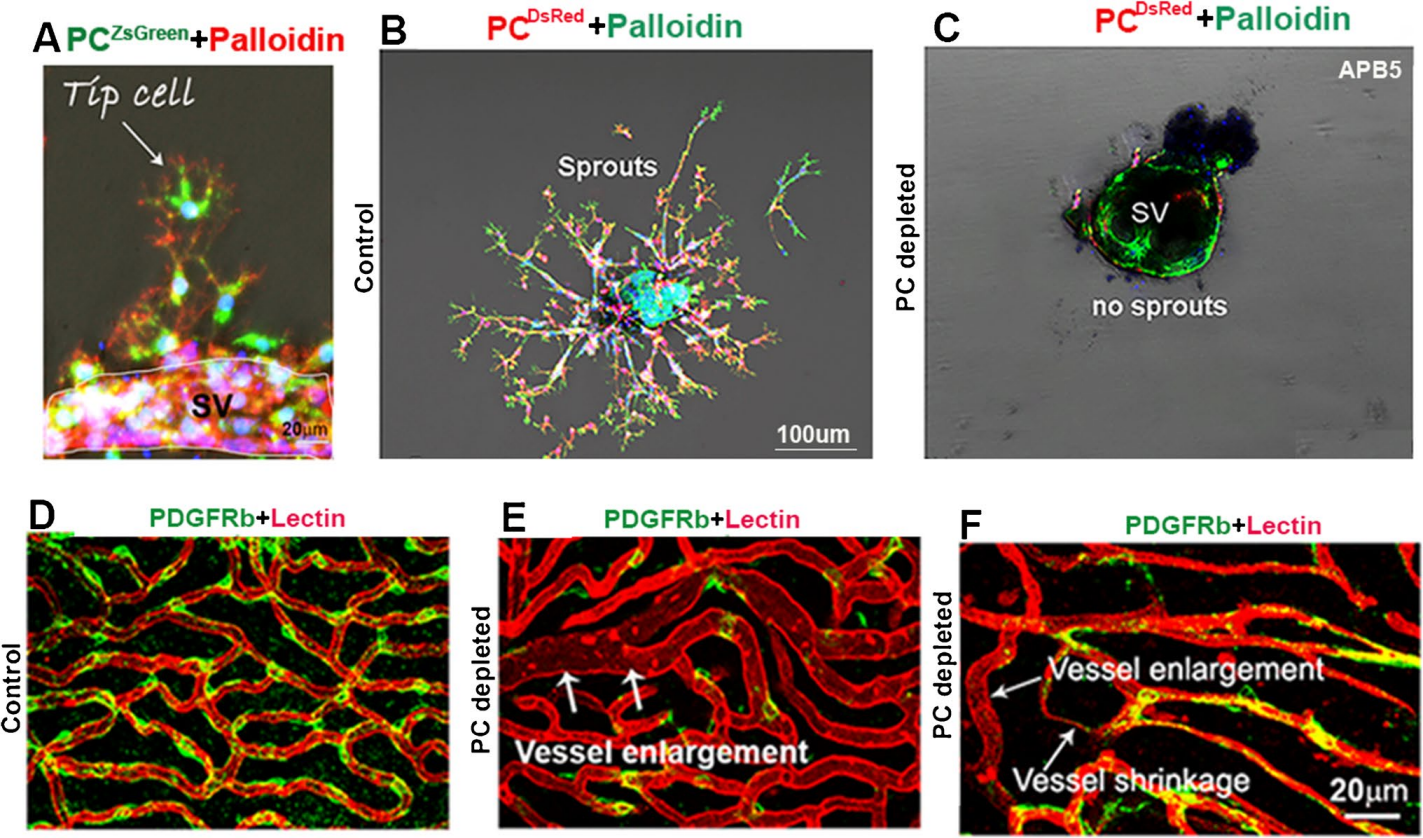
**A** Confocal images show NG2 + PCs in green, acting as leading tip cells (indicated by an arrow) during angiogenesis. The tissue explant was obtained from an adult NG2 ZsGreen mouse. **B** Angiogenesis occurring in a strial explant from an NG2 DsRed mouse cochlea, five days after treatment with VEGF-A165. **C** The depletion of PCs using APB5, an anti-PDGFRβ antibody, significantly impairs angiogenesis. **D** The normal distribution of PCs in a non-PC-depleted control animal. **E**, **F** Both panels show that PC depletion leads to either vessel enlargement (as indicated by the arrows in **E**) or shrinkage (as indicated by the arrows in **F**)

PCs, also known for their remarkable stem cell potential, possess the ability to proliferate new PCs and promote angiogenesis under vascular growth factor stimuli [20]. Using an in vitro three-dimensional tissue explant model, Wang et al. [20] have shown that strial progenitor cells can promote the formation of new vascular sprouts (see Fig. 5A and B). In contrast, damage to PCs in vitro (Fig. 5C) or conditional depletion of strial PCs in vivo leads to a halt in the formation of new sprouts and results in cochlear vascular degeneration [19], as shown in Fig. 5E and F. Mechanistically, recent RNA sequencing of cochlear strial PCs has revealed their ability to release various growth factors including vascular endothelial growth factor (VEGF) that regulate angiogenesis [21].

Generally, the functions of PCs are linked to their anatomical location and transcriptional characteristics [5]. Recent single-cell RNA sequencing of strial PCs has revealed the existence of two subclasses of PCs within the vascular network of the SV, each distinguished by unique signature genes [5]. One type of PCs is characterized by the presence of α-smooth muscle actin (αSMA) and transgelin (TAGLN). These cells are predominantly located around pre-capillaries, post-capillaries, and near true capillaries. In contrast, another type of PCs does not express TAGLN and is primarily found in the regions of true capillaries, where they are enriched with transporter genes, such as Abcc9 and Kcnj8. TAGLN encodes a protein that is important for cytoskeletal remodeling and contractility, indicating that TAGLN positive PCs may function as contractile sphincters that work in conjunction with vascular smooth muscle to regulate capillary blood flow according to metabolic demands. On the other hand, Kcnj8 PCs may play a role in mediating the exchange of substances from the blood. However, the specific phenotypes of cochlear PCs and how these distinct phenotypes relate to various vascular functions across different regions of the cochlea are not well-studied and remain poorly understood.

PCs are largely believed to originate from embryonic mesenchyme and the ectoderm-derived neural crest [22]. While some studies have shown they can also be tans-proliferated from adult tissue-resident mesenchymal stem cells [23] and glial populations [24].

**Fig. 6:**
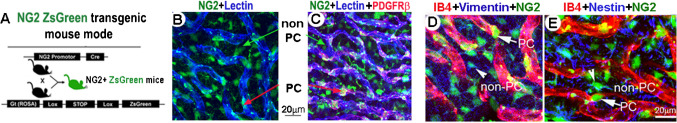
NG2 + PCs populate the vessel wall, while NG2 + non-PCs reside in the perivascular zones of the stria vascularis. **A** Creation of the Cre-NG2 + ZsGreen transgenic mouse model. **B** Confocal images show NG2 + cells on strial capillaries (red arrow) and around the stria vascularis (green arrow). **C** NG2 + cells on vessel walls are identified as PCs based on PDGFRβ positivity (red arrow), while those in the perivascular niche do not express PDGFRβ but are positive for stem cell markers vimentin (**D**, arrowhead) and nestin (**E**, arrowhead)

**Fig. 7:**
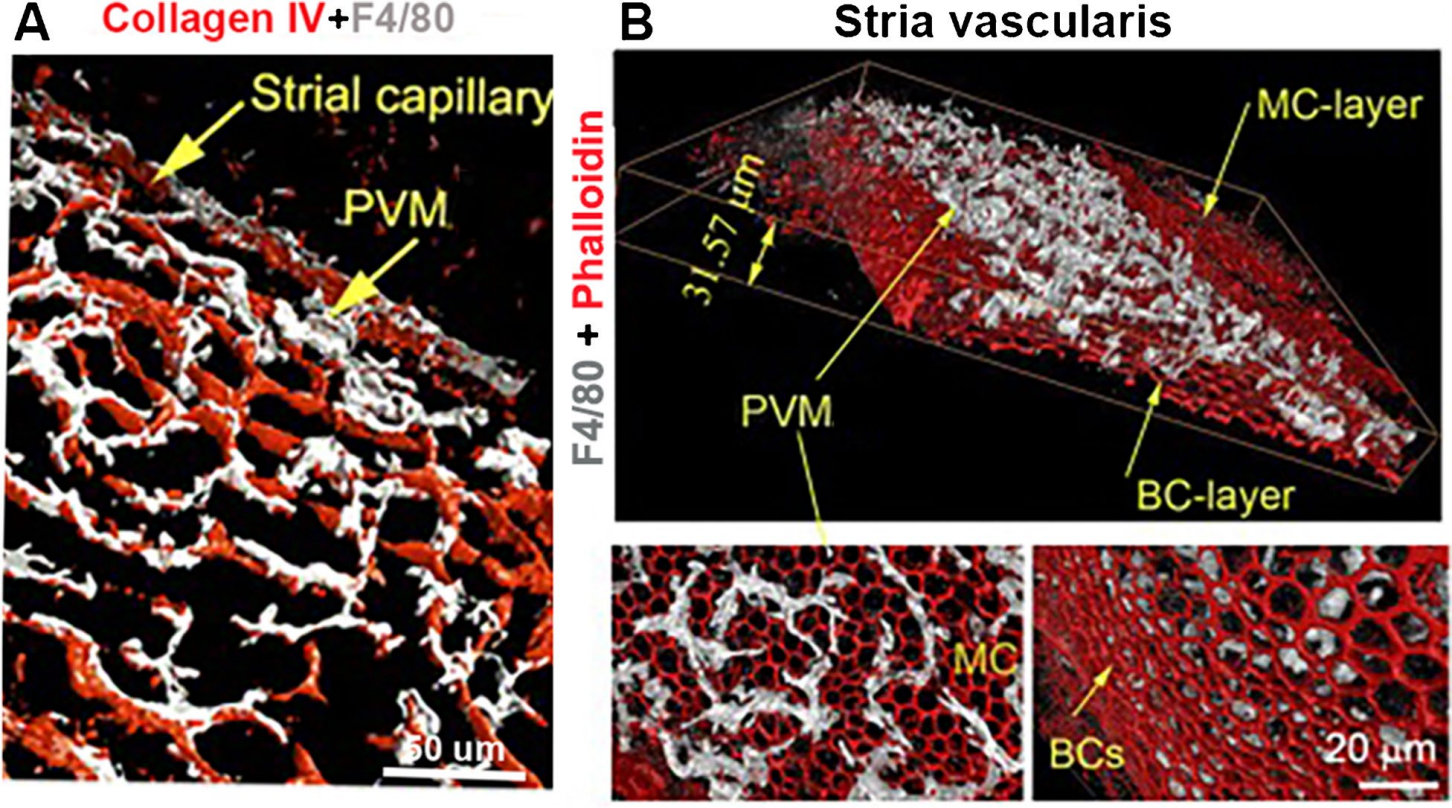
**A** The 3D rendered confocal stack illustrates the ramified structure of the PVMs, which are closely associated with capillary walls (indicated by arrows). **B** (upper) A 3D confocal image of the stria vascularis shows PVMs positioned between the marginal cell (MC) and basal cell (BC) layers (pointed by arrows). **B** (lower) Different angles reveal that PVM processes are found near subepithelial marginal cells and have less contact with basal cells (arrow)

In the SV of the adult cochlea, a subset of unclassified perivascular neural/glial antigen 2 (NG2)-expressing cells have long been found surrounding the strial capillary network of the adult cochlea. This population was initially observed in a typical NG2-CreERT2 knock-in mouse model (Wang et al., 2019), where Cre activity was activated at the embryonic stage, resulting in persistent NG2 + signaling after birth (Fig. 6B-E). Postnatally, these cells no longer express NG2 after birth. They also do not express the PC marker PDGFR-β [19], but they do, however, express mesenchymal stem cell markers such as vimentin and nestin [25, 26], as shown in Figs. 7D and E. Early studies have shown that NG2 + PCs play a vital role as tip cells in cochlear angiogenesis in adult mice (Wang et al., 2019). However, the source of precursor PCs remains elusive. Recent research indicates that postnatal mesenchymal stem cells are located in perivascular niches and function as vascular progenitor cells [27]. These cells are essential for repairing damaged blood vessels throughout life [28]. It would be intriguing to investigate whether the prenatal NG2-expressing cells found in perivascular niches are progenitors of PCs. Future research into the origins and sources of PCs in adults is crucial for understanding how the stability of adult vasculature is maintained and for developing strategies to repair damaged and aging endothelium by targeting PC progenitors.

### PVMs, a Type of Specialized Tissue-Resident Macrophage, Are Closely Located at the Interface of the Marginal Cell-Endolymph and Patrol the ISFBB

#### PVM Location

Tissue resident macrophages are immune cells widely distributed across various organs and tissues in mammals and the human body, playing a crucial role in the onset and progression of diseases [29]. PVMs are a specialized subset of tissue-resident macrophages found within the cochlear SV of mammals including mouse [1], monkey [30], and human cochlea [31, 32]; where they maintain close associations with blood vessels, as shown in Fig. 7A [33]. These macrophages have also been reported in various other organs, including the brain, skin, liver, and retina [34, 35]. Specifically, cochlear PVMs are situated within the perivascular spaces between the marginal cell and basal cell layers of the SV (Fig. 7B, upper). They intriguingly are located in or beneath the subepithelial marginal cells (Fig. 7B, lower) and closely associated with vascular networks [1, 36, 37]. They express an array of macrophage markers such as F4/80, CD68, Iba1, and CD11b, along with scavenger receptors A1 (SR-A1) and B1 (SR-B1) [1].

#### PVM Ontogeny

**Fig. 8:**
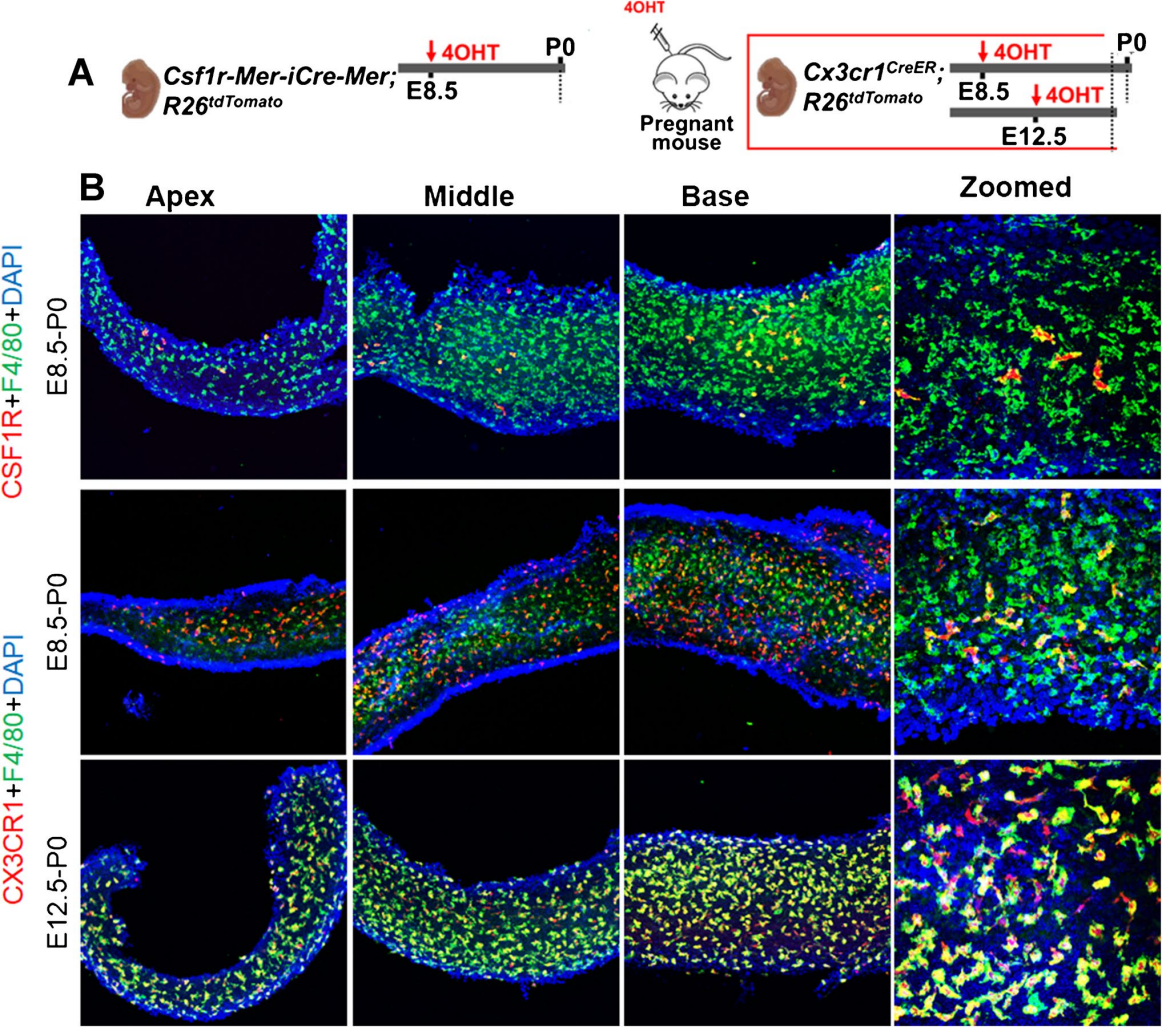
**A** Fate mapping of the experimental setup illustrates the timing of 4OHT injections that induce Cre recombination, resulting in tdTomato expression in PVMs (shown in red). **B** Representative confocal images demonstrating PVMs (red) are of embryonic origin, primarily originated from both yolk sac at E8.5 and the fetal liver at E12.5. *4OHT* hydroxytamoxifen

Tissue-resident macrophages largely originate from embryonic sources and in many cases self-maintain independently without monocyte input [38]. However, in certain tissues, monocyte-derived macrophages replace these over time or because of tissue injury and inflammation. Recent studies indicate that PVMs are seeded in the SV during waves of embryonic hematopoiesis [39]. Study indicates that certain populations arise from the yolk sac through Csf1r-dependent signaling and express marker proteins such as F4/80, Iba1, and CD68. In contrast, another subtype develops from the fetal liver via a Csf1r-independent mechanism, characterized by a CD11b + phenotype [39, 40]. Consistent with the reports, our recent fate-mapping studies, employing two macrophage-specific Cre driver mouse models, indicated that PVMs arise from both the fetal liver and the yolk sac, as illustrated in Fig. 8B. During adulthood, the embryonic PVMs are continuously replenished by monocytes from the bone marrow (BM), as noted in earlier research [1]. However, the exact nature of the embryonic progenitors that give rise to adult tissue-resident macrophages, and the mechanisms enabling macrophage population maintenance in adults are still not well studied.

The characteristics of PVMs in C57BL/6 J mice are particularly unique. In our early research, we referred to them as PVM/M (macrophage-like melanocytes) due to their significant melanin content and the expression of GST alpha 4, a protein that is integral to glutathione peroxidase activity [33]. Recent review suggests that the melanin observed in PVMs may originate from their phagocytic activity directed towards cellular debris and components derived from melanocytes situated within the SV. This process involves the engulfing and processing of melanocyte fragments, potentially leading to the accumulation of melanin within the PVMs [36]. An intriguing question persists: why do PVMs maintain a significant quantity of melanin following extended in vitro culture in the absence of melanocytes? A few recent reviews from non-auditory organs mentioned “melano-macrophages” in cold-blooded species, which are pigmented phagocytic cells crucial for fish immune responses [41–43]. These cells are found in the kidneys, liver, and spleen. Notably, melano-macrophages in the liver are primarily located adjacent to blood vessels or bile ducts in the tissues of Atlantic salmon [42]. These melano-macrophages contain hemosiderin and lipofuscin, which are involved in melanin production [44]. An important question arises: Are perivascular macrophages (PVMs) in C57BL/6 J mice a type of “melanophage”? To determine this, specific studies are needed to clarify whether the melanin found in PVMs is produced internally or derived from external sources in vivo*.*

Another question that arises is: What is the potential relationship between PVMs and melanocytes? Melanocytes, which are often referred to as intermediate cells, play a crucial role in generating the EP, which is essential for sound transduction in sensory hair cells. Early studies suggest that there are two types of intermediate cells: one that appears light and another that appears dark [45]. Some of these intermediate cells contain lysosomes formed through autophagic or endocytic activity, corresponding to acid phosphatase-positive inclusions that have been previously reported [46]. A study by Conlee et al. in 1989 showed that pigmented cells were primarily located adjacent to the strial capillaries. Ultrastructural studies of the stria in pigmented cats revealed that these perivascular cells often contained a high abundance of pigmented organelles and other distinguishing structural features that set them apart from typical intermediate cells [47]. Are PVMs a type of dark intermediate cell that has been previously described?

Melanocytes originate either from the neuroepithelium of the optic cup or from the neural crest. Recent studies indicate that cochlear intermediate cells arise from both melanoblast and Schwann cell precursors [48]. Interestingly, some research suggests that the myeloid cells in ectothermic vertebrates could be classified as melanocytes [42]. Lineage tracing has shown that early PVMs are indeed myeloid cells (see Fig. 8). Current advances on cochlear intermediate cells have provided valuable insights into these cell types, revealing that they express several important genes, including endothelin [49], Sox10 [50], and Pax3 ([51]. Notably, these genes are also linked to the development and function of macrophages [52–54]. Nevertheless, to clarify the relationship between PVMs and melanocytes and their anatomical interactions within the stria vascularis, specific immune labeling of these cell types is necessary. Particularly imaging the stria vascularis at the nanoscale using focused ion beam scanning electron microscopy (FIB-SEM) will aid in identifying the intricate structures among the different cell types within the stria vascularis.

## PVM Physiological Role

The physiological role of PVMs in the SV and their impact on hearing are not yet fully understood. The available related information in the cochlea is quite sparse. However, recent research has shown that macrophages are organ-specific and strongly associated with both organ function and the microenvironment, influencing their roles during both developmental stages and steady states [55]. Specifically, studies indicate that the functions of PVMs are influenced by their tissue location and various environmental factors [56]. Macrophages, in general, are strategically located in specific regions of tissues to effectively monitor signs of infection, harmful agents, or injury. As immune cells, they help clear debris and may also contribute to the survival and repair of damaged tissue [57]. In the stria, PVMs closely interact with marginal cells, as shown in Figs. 7A and B [33]. Recently, Liu et al. (2024) proposed that PVMs extend slender processes between marginal cells, potentially acting as immune sensors that monitor the condition of the endolymph [58]. The ion gradients in the scala media are critical for the transduction of sensory hair cells. PVMs are located at the boundary of the marginal cell-endolymph layer, suggesting they may serve as gatekeepers for endolymph homeostasis and immunity. In fact, evidence from studies on the central nervous system suggests that PVMs interact directly with cerebrospinal fluid (CSF) to help clear metabolic waste and regulate the dynamics of CSF flow [59]. In the cochlea, PVMs also patrol the interstitial tissue-blood barrier, which is the interface between the bloodstream and the surrounding strial tissues. This positioning enables them to effectively monitor, detect, and respond to any changes or issues within blood circulation. Study shows brain PVMs clear vascular deposits such as vascular amyloid Aβ, which is a harmful material that accelerated the local neurovascular dysfunction [60].

### Interaction Between PVMs and ECs

Research in non-auditory indicate that the interaction between ECs and PVMs is essential for various processes, including vascular permeability, angiogenesis, blood flow regulation, inflammation, and tissue repair in many organs [61]. However, how cochlear PVMs interact with ECs to regulate vascular permeability, angiogenesis, and the repair of damaged ECs is little to know.

## PVM in Vascular Permeability

An earlier study by Zhang et al. (2012) found that the depletion of perivascular macrophages (PVMs) leads to a compromised endothelial barrier in the stria vascularis (SV), as evidenced by a decrease in the expression of tight junction (TJ) proteins [33]. Recent reviews on non-auditory organs emphasize that PVMs play a crucial role in stabilizing TJs between endothelial cells (ECs), thus limiting vascular permeability [62]. For instance, research has shown that the depletion of microglia in the brain significantly increases vascular leakage during hypoxia, which is correlated with astrocyte-vascular uncoupling and a loss of TJ proteins [63]. Similarly, studies involving mesenteric and dermal tissues have demonstrated that depleting macrophages using clodronate liposome treatment markedly enhances vascular permeability through mechanisms related to VE-cadherin [64]. However, recent findings by Hirose et al. (2019) suggest that the depletion of cochlear PVMs does not affect the permeability of the blood-perilymph barrier [65]. Clearly, further investigations are needed to elucidate the precise roles and underlying molecular mechanisms of PVMs in maintaining the integrity of vascular permeability in the cochlea.

## PVMs in Angiogenesis

Studies have also highlighted the significant role of PVMs in stimulating the formation of new blood vessels through the secretion of pro-angiogenic factors such as VEGF-A, TGF-β, and IL-1β, along with various proteases that promote vessel growth [66, 67]. During angiogenesis, macrophages release pro-angiogenic factors, such as VEGF-A, along with various proteases like matrix metalloproteinases (MMP-2, MMP-7, MMP-9, and MMP-12). These substances aid in the release of pro-angiogenic growth factors that are embedded within the perivascular matrix, thereby promoting vascular growth [67]. However, the specific roles of macrophages in cochlear angiogenesis and their participation in the repair of vascular damage remain largely underexplored. An earlier study by Neng et al. (2015) utilizing an in vitro cell line model demonstrated that cochlear PVM stabilizes endothelial tube formation and enhances the tube stability [68].

## PVMs in Blood Flow Regulation

Beyond their role in angiogenesis, recent evidence suggests that PVMs are crucial for regulating blood flow and facilitating the infiltration of immune cells following tissue damage [69]. For instance, a study conducted by Vagesio et al. (2021) demonstrated that PVMs accumulate around blood vessels at injury sites and help regulate blood flow in muscles through nitric oxide signaling [70]. However, it remains to be determined whether cochlear PVMs play a role in blood flow regulation. In contrast, a recent study by Yi et al. (2025) demonstrated that cochlear ECs negatively regulate macrophage activity through the CX3CL1-CX3CR1 pathway during noise-induced oxidative stress events [71].

## Vascular Degeneration and PVM Activation in ARHL

ARHL, or presbycusis, is a gradual and irreversible decline in auditory sensitivity [72]. It can be classified into three phenotypes: metabolic, neural, and sensory [73, 74]. Metabolic presbycusis is thought to be linked to strial atrophy [73, 75]. Recent studies have highlighted that a reduction in capillary volume and the activation of PVMs are early indicators of cochlear degeneration as we age [37, 68].

### EC Senescence, PC Loss, and Endothelial Leakage

**Fig. 9:**
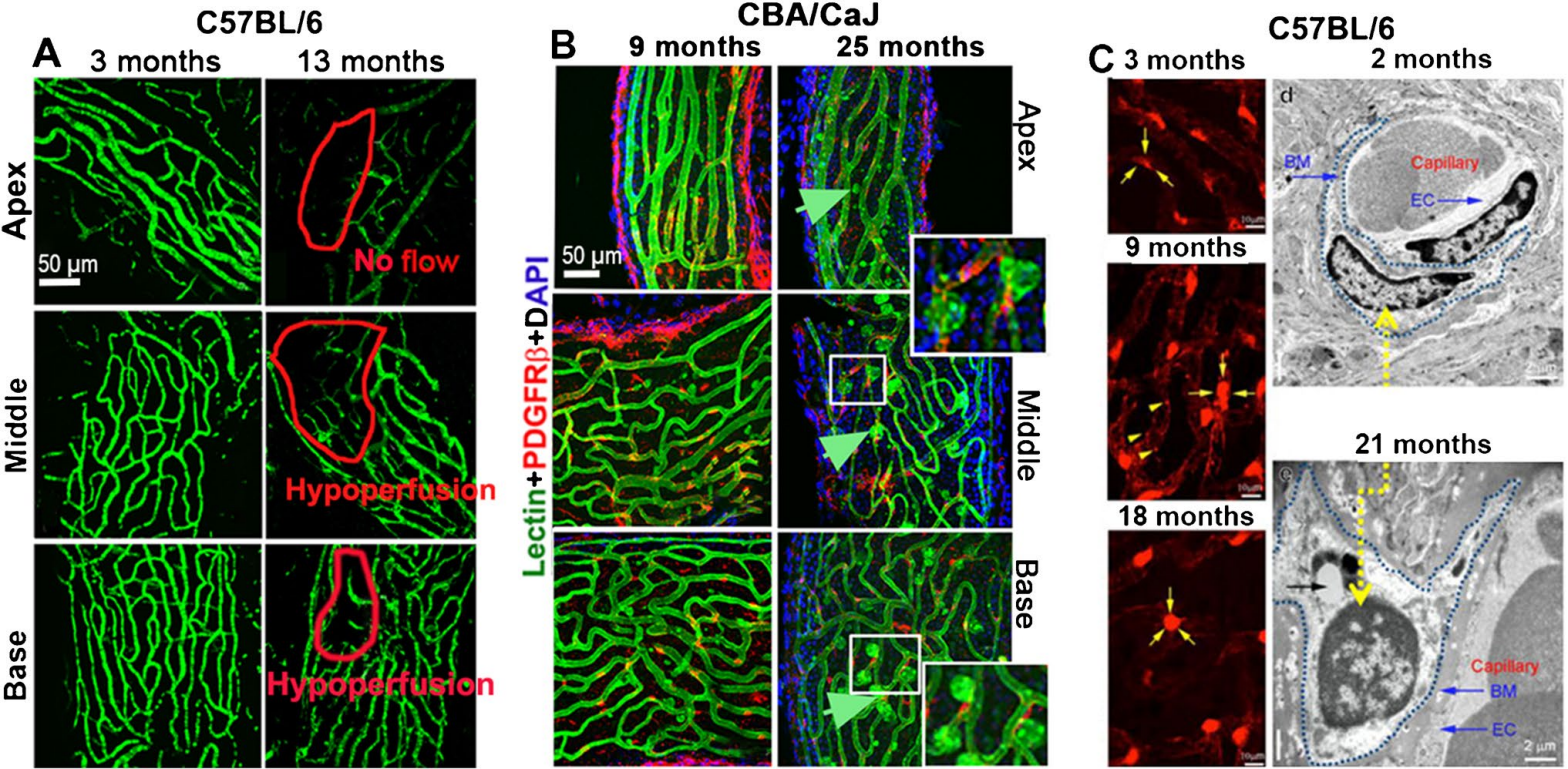
Vascular degeneration in aging cochleae. **A** Aged C57BL/6 mice exhibit low blood perfusion, as indicated by the absence of EGFP-tagged blood cells in circulation (red circles). **B** Aged CBA/CaJ mice show signs of vascular shrinkage and leakage, as highlighted by green lectin staining (arrows and square indicate affected areas), in contrast to the vascular structure seen in younger animals (**B**, left). **C** In young C57Bl/6 mice, PCs are flattened and closely associated with blood vessels (arrows, upper left). In contrast, aged mice display significant protrusion and migration of PCs (arrows, middle and lower). The separation of PCs from endothelial cells (EC) with disruption of the basement membrane (BM) is more pronounced in older animals compared to younger ones, as shown by transmission electron microscopy (TEM, yellow arrows)

As we age, blood vessels naturally regress, becoming fragile and permeable with reduced blood perfusion, as shown in Fig. 9A. Vascular aging is generally characterized by structural and functional changes within the vascular system, which include increased vessel stiffness, thickening of vessel walls, and the formation of non-functional spaces within the vessels [76].

Age-related degeneration of the endothelium is characterized by the accumulation of senescent ECs and a decline in their functional phenotype [77]. EC senescence refers to the gradual decline in the proliferation and differentiation abilities, as well as the physiological functions of these cells as they age [78]. Although the mechanisms driving this degeneration are not yet fully understood, EC senescence is believed to be linked to oxidative stress, chronic inflammation, and alterations in the extracellular matrix that impact PC attachment and functionality [79]. Early research by Thomopoulos (1997) shows degeneration of cochlear ECs and PCs in the gerbil [80]. A recent study by Neng et al. (2015) demonstrates a strong correlation between reduced vascular volume and a diminished population of PCs [68]. Findings revealed that, in aged animals, the protrusions of PCs exhibited decreased contact with ECs, along with signs of vacuolization in ECs in C57BL/6 mice, as shown in Fig. 9C [68]. Similar pathologies, including the loss of PCs, vascular shrinkage and leakage have also been observed in aged CBA/CaJ mice, a frequently used animal model for studying ARHL [81], as illustrated in Fig. 9B (right panels).

In addition to the degeneration of PCs and ECs, there is a notably thickening of the basement membrane in the aging SV [80]. This phenomenon has been observed in both aged animal models and in human temporal bones sourced from older adults [82, 83]. Interestingly, a recent study has found that PCs can transform into fibroblasts as a result of aging [84]. These PCs synthesize and deposit extracellular matrix components, such as collagen and other structural proteins, which contribute to tissue fibrosis typically observed in aged skin. Additionally, earlier research demonstrated that PCs are involved in fibrous tissue deposition in aged skeletal muscle [85]. However, it remains unclear whether similar changes in the PC phenotype occur in the aging cochlea and how these alterations might contribute to the thickening of the basement membrane during the aging process. Notably, cochlear PCs have been observed transforming into fibroblasts in response to noise exposure in the stria [86].

The reduction in vascular volume, loss of PCs, and thickening of the basement membrane adversely affect blood flow to the cochlear lateral wall. This, in turn, impacts the energy supply and maintenance of the EP. Early studies from various laboratories have reported decreases in blood flow and declines in EP in aged animals [87, 88]. These changes could be significant contributors to the decreased auditory sensitivity often associated with aging.

### Dysfunction of PVMs Contributes to Chronic Inflammation

Chronic inflammation appears to be a prevalent characteristic of age-related neurodegeneration across various organs including ARHL, as highlighted in recent reviews [89–91].

The “immunologic theory of aging,” first articulated by Walford in 1969, posits that chronic inflammation, aptly termed inflammaging, plays an integral and detrimental role in the aging process [92]. Recent reviews emphasize that aging is closely associated with persistent, low-grade chronic inflammation, often referred to as sterile inflammation. This type of inflammation significantly impairs tissue function over time.

**Fig. 10:**
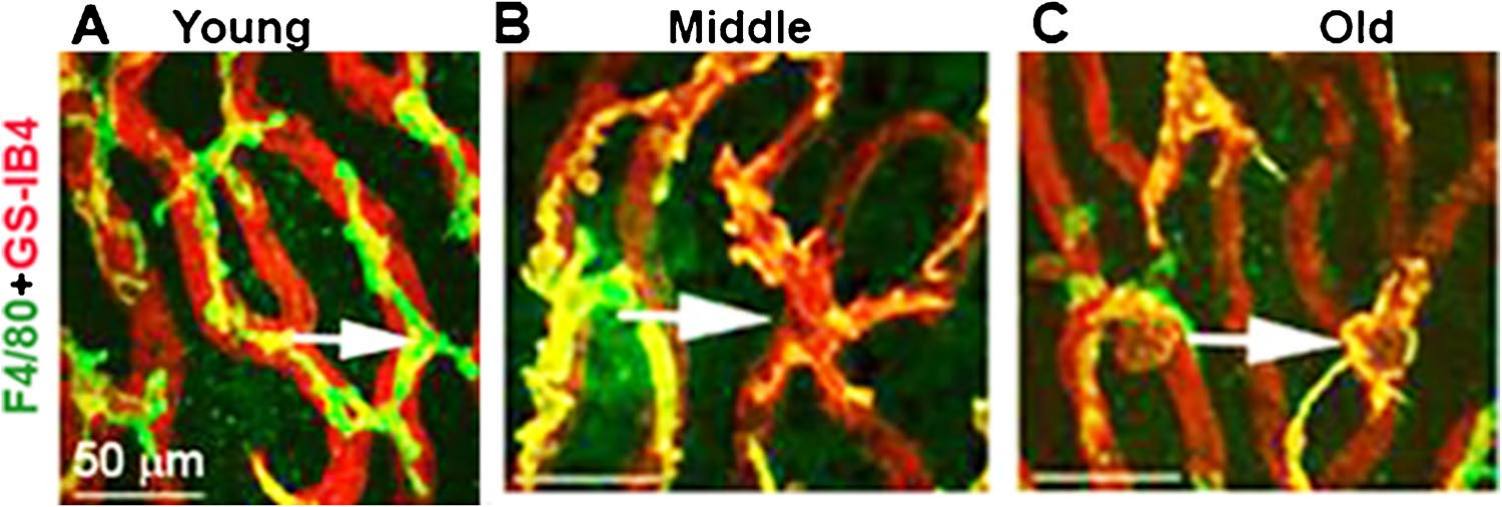
**A**–**C** Morphological alteration of PVMs with aging. In young animals, PVMs have long, branched processes alongside capillaries (young, **A**), but with aging, these processes retract (middle age, **B**), resulting in a flatter, amoeboid appearance (old, **C**)

At the cellular level, the mechanisms driving macrophage-related tissue inflammation during aging are closely associated with immunosenescence [93]. This phenomenon is characterized by a reduced ability of the immune system to respond effectively to both internal and external antigens [94]. As a result, there is insufficient clearance of senescent cells in aged tissues. Typically, dysfunctional macrophages in aged organs exhibit noticeable morphological and functional changes [95]. An early study by Neng et al. (2015) has demonstrated the significant morphological differences in cochlear PVMs between young and aged animals [68]. In younger C57BL/6 J mice, PVMs typically display long, branched processes that weave gracefully alongside strial capillaries as shown in Fig. 10A. However, as these mice get older, specifically at 6, 9, and 12 months, certain PVMs begin to retract their intricate processes (Fig. 10B). By approximately 21 months, many of these macrophages adopt a flattened, amoeboid shape, exhibiting markedly reduced contact with capillaries (Figs. 10C). Accompanying these striking morphological alterations, notable biochemical changes occur in aging PVMs. In older animals, terminal galacto-pyranosyl groups, which are indicative of macrophage activation, become prominently exposed on membrane surfaces, rendering them detectable via lectin GS-IB4 staining [96]. Recently, research by Lang et al. (2023) further highlighted a strong correlation between increased PVM activation and cochlear inflammation, particularly in the context of ARHL [37]. They noticed macrophage activation within the SV in middle age or possibly earlier, continuing in an age-dependent manner as aging progresses [37]. At the molecular level, activated macrophages produce pro-inflammatory cytokines, including granulocyte colony-stimulating factor (G-CSF), macrophage colony-stimulating factor (M-CSF), granulocyte–macrophage colony-stimulating factor (GM-CSF), interleukin (IL)−1, IL-3, IL-6, TNF-α, interferons (IFNs), and Flt3 ligands. These factors drive cell damage and senescence in aging tissues [94]. Studies from other labs have also shown that inflammatory mediators such as C-reactive protein, interleukin-6 (IL-6), and TNF-α are elevated in aged cochleae [97, 98]. Transcriptomic comparison analysis of differentially expressed genes in the cochlea of young and aged C57BL/6 mice revealed significant upregulation of inflammatory genes [99]. Despite these advances, the specific molecular pathways driving chronic inflammation in the cochlea are still not completely defined.

## Conclusions and Future Perspectives

The vascular system and the innate immune system in the SV are closely integrated and both play crucial roles in maintaining cochlear homeostasis, which is essential for normal hearing. Within the stria, specialized ECs form a tightly regulated ISFBB. PCs and PVMs serve as secondary support and defense mechanisms within this barrier. PCs are vital for maintaining vascular integrity, while PVMs monitor the microenvironment. The interactions between PCs, ECs, and PVMs create a complex system that is essential for sustaining strial hemostasis. However, how these individual cells and their interactions mediate strial health and degeneration, as well as the molecular signals involved, are not well understood at this stage. Gaining a deeper understanding of the vascular-immune interactions within the cochlea and therapeutically targeting both systems may help alleviate ARHL, which significantly impacts the quality of life in older adults and potentially increases the risk of cognitive decline.

## Data Availability

Not applicable.
